# Social network characteristics and cognitive function, decline, and mortality: A joint modeling approach

**DOI:** 10.1002/alz.71600

**Published:** 2026-07-12

**Authors:** Ruijia Chen, Alexander Ivan B. Posis, Anna M. Pederson, Jingxuan Wang, Andrew K. Hirst, L. Paloma Rojas‐Saunero, Ye Ji Kim, Sebastien Haneuse, Rachel Peterson, Dan Mungas, Jacqueline M. Torres, Elizabeth Rose Mayeda, Paola Gilsanz, Rachel A. Whitmer, Maria M. Corrada, M. Maria Glymour

**Affiliations:** ^1^ Department of Community Health Sciences UCLA Fielding School of Public Health, University of California, Los Angeles Los Angeles California USA; ^2^ Department of Public Health Sciences University of California, Davis Sacramento California USA; ^3^ Department of Epidemiology Boston University School of Public Health Boston Massachusetts USA; ^4^ Department of Epidemiology Harvard T.H. Chan School of Public Health Boston Massachusetts USA; ^5^ Division of Research Kaiser Permanente Northern California Pleasanton California USA; ^6^ Department of Epidemiology, UCLA Fielding School of Public Health University of California, Los Angeles Los Angeles California USA; ^7^ Department of Biostatistics Harvard T.H. Chan School of Public Health Boston Massachusetts USA; ^8^ School of Public and Community Health Sciences University of Montana Missoula Montana USA; ^9^ Alzheimer's Disease Research Center University of California, Davis Sacramento California USA; ^10^ Department of Epidemiology and Biostatistics University of California, San Francisco San Francisco California USA; ^11^ School of Medicine University of California, Irvine Irvine California USA

**Keywords:** 90+, cognitive decline, cognitive function, mortality, social network

## Abstract

**INTRODUCTION:**

Maintaining social relationships is challenging in advanced old age due to frequent changes in social networks. Little is known about whether social networks are associated with cognitive function, decline, and mortality in this population, or whether these associations differed by sex/gender.

**METHODS:**

Participants aged ≥90 years (*n* = 677; mean age = 92.9, SD = 2.2) from the multi‐ethnic LifeAfter90 Study completed cognitive testing every 6 months (2019–2022). Joint models examined associations of social networks with cognitive decline and mortality, overall, and by sex/gender.

**RESULTS:**

Over 1.63 years of follow‐up, 163 (24%) participants died. Baseline structural and functional social network characteristics were associated with higher executive function and verbal episodic memory, with consistent findings across sex/gender. Social networks did not predict cognitive decline or mortality.

**DISCUSSION:**

Among adults aged 90+, social networks were linked to better cognitive function but not to short‐term cognitive decline or mortality.

## Highlights

- In this racially and ethnically diverse population of older adults age 90+, social networks were associated with better performance in executive function and verbal episodic memory tests.
- The associations of social networks with levels of cognitive function were generally consistent between women and men, although the estimates were less precise among men.
- Social networks were not associated with cognitive decline or mortality among older adults age 90+.

## 1 BACKGROUND

Older adults aged 85 years or older are the fastest‐growing age group in the United States.^1^, ^2^, ^3^ They experience high rates of morbidity, functional disability, and mortality, and their social networks are frequently reduced in size due to illness and bereavement.^4^, ^5^, ^6^ Factors typically associated with cognitive health outcomes and mortality may not apply to the oldest‐old, and in some cases, may even operate in the opposite direction.^3^, ^7^ For example, Corrada et al. examined the associations between the age of hypertension onset and risk of dementia in the 90+ Study and found that developing hypertension at older ages may protect against dementia. This finding contrasts with associations observed in younger older adults and was robust to survival bias.^7^ Investigating modifiable risk factors associated with cognitive functioning and mortality among the oldest‐old has important public health implications. Such findings will inform our understanding of cognitive functioning and survival across all ages and guide the development of strategies to reduce disease burden as the population aged 90 years and older continues to grow.

Social networks may be modifiable factors that influence cognitive health outcomes and mortality. They may provide important resources for the health and well‐being of adults in advanced old age. Structural features of social networks include size and composition (e.g., the ratio of friends to family members in the network). In contrast, functional features of social networks correspond with network quality, such as emotional support provided by network members. Structural characteristics of social networks are thought to affect health outcomes by shaping access to information and opportunities that promote health behaviors, whereas functional social networks may be more likely to improve health by buffering the harmful effects of stressors.^8^ Given the potentially different health effects, structural and functional characteristics of social networks should be examined separately. Despite mixed evidence, research generally suggests that both structural and functional aspects of social networks are associated with better cognitive functioning and lower mortality risk.^8^, ^9^, ^10^ However, most existing research focuses on middle‐aged or early late‐life populations; far less is known about social networks and cognition or mortality among those in advanced old age.^8^, ^9^, ^11^, ^12^

Social network characteristics and their associations with health outcomes often differ by gender.^13^, ^14^, ^15^, ^16^ Compared with men, women tend to report more diverse and supportive networks and rely less on familial ties.^13^, ^14^, ^16^ As women are more likely to invest in cultivating and maintaining social relationships, they may be more vulnerable to the adverse health effects of limited or unsupportive networks. In contrast, men tend to rely more on spousal or family networks and may be particularly vulnerable when these networks are absent.^17^ Although relatively few studies have examined gender‐specific associations between social networks and cognitive function or mortality, most report stronger associations among older women than among men.^18^, ^19^, ^20^ Whether this gender difference persists into advanced old age remains unclear. Clarifying these patterns is important for identifying population subgroups most likely to benefit from social network‐based interventions to promote cognitive health and longevity.

Identifying factors associated with cognitive outcomes and mortality among adults in advanced old age requires complex modeling techniques, given the high rate of death and attrition in this population. Both social networks and cognitive function may influence mortality risk and neglecting differential survival can bias the estimated associations of social networks with cognitive decline. Joint models, which allow for the simultaneous modeling of cognitive trajectories and the hazard of mortality or attrition, have been shown to provide estimates that are robust to survival bias when correctly specified.^21^

This study aims to characterize the associations of structural and functional aspects of social networks with cognitive function, decline, and all‐cause mortality in the LifeAfter90 (LA90) Study, a racially and ethnically diverse cohort of adults aged 90 years and older. We hypothesized that (1) higher levels of structural and functional social networks would be associated with higher levels of executive function and verbal episodic memory, as well as a slower rate of decline in these domains; (2) higher levels of structural and functional social networks would be associated with a lower hazard of mortality; and (3) these associations would be stronger among women than among men.

**RESEARCH IN CONTEXT**

1. **Systematic review**: The authors searched PubMed to identify studies on associations of social networks with cognitive function and mortality. Although social networks and cognitive function have been widely studied, few studies included individuals aged 90+. Little is known about whether late‐life social networks are associated with cognitive function, decline, and mortality in this population.
2. **Interpretation**: Both structural and functional aspects of social networks were associated with better performance on executive function and verbal episodic memory tests in this sample of older adults aged 90+. These associations were generally similar between older women and men, though estimates were less precise for men. Neither structural nor functional aspects of social networks were associated with cognitive decline or mortality risk in the overall sample or when stratified by sex/gender.
3. **Future directions**: Further research is needed to determine whether these associations are replicable across diverse populations and when using alternative social network measures.

## 2 METHODS

### 2.1 Data

The LA90 Study is an ongoing longitudinal cohort that aims to investigate lifecourse determinants of dementia incidence, cognitive decline, neuropathologic changes, and brain imaging markers in a racially and ethnically diverse cohort of individuals aged 90 years and older.^22^ Participants are long‐term members of Kaiser Permanente Northern California (KPNC), aged 90 years or older, and residing in the San Francisco Bay and Sacramento areas of California. Eligible individuals were KPNC members at some point between 1964 and 1992 and spoke either English or Spanish. Exclusion criteria included a diagnosis of dementia or other neurodegenerative disease, hospice care, or dialysis at the time of enrollment (as indicated in the electronic medical record), or inability to provide informed consent. Study enrollment began in July 2018 and is ongoing. Data collection occurred every 6 months through in‐person interviews, with a shift to phone interviews following the onset of the coronavirus disease 2019 (COVID‐19) pandemic in March 2020. Social network information was first collected during the second wave, which included 757 participants and served as the baseline for this analysis. For the present study, we included data collected through December 2022. Approximately 10% of covariates, exposures, and outcomes were missing at baseline. We assumed the data were missing at random and restricted the analytical sample to those with complete data on social networks, covariates, and cognitive function scores at baseline, which resulted in an analytical sample of 677 participants. The study was approved by the KPNC and University of California, Davis, institutional review boards, and all participants provided written informed consent.

### 2.2 Measures

#### 2.2.1 Structural characteristics of social network

*Social network size* was determined by the total number of living children, relatives, and friends with whom participants felt close. Specifically, participants answered three separate questions about the number of living children, relatives, and friends they considered close. Responses to these questions were summed to create a composite social network size score, with higher scores reflecting larger social networks. We capped the social network size at the 99th percentile to reduce the influence of extreme values.

*Social network composition* captured the relative importance of friends compared to family members (i.e., spouse, living children, and relatives) within an individual's social network. It was calculated by dividing the number of friends by the total number of people in the social network, then multiplying by 100. A higher score indicates a greater proportion of friends, while a lower score indicates a greater proportion of family members.^23^

#### 2.2.2 Functional characteristics of social network

*Perceived emotional support* was assessed by questions from the National Institutes of Health Toolbox Adult Social Relationship Scale.^24^ Participants were asked to indicate how often they: (1) have someone who understands their problems; (2) have someone who listens to them when they need to talk; (3) feel there are people they can talk to if they are upset; (4) have someone to talk with when they have a bad day; (5) have someone they trust to talk with about their problems; (6) have someone they trust to talk with about their feelings; (7) can get helpful advice from others when dealing with a problem; and (8) have someone to turn to for suggestions about how to deal with a problem. The participants rated each item on a 5‐point Likert scale ranging from 1 (never) to 5 (always). We summed the scores for all items to obtain a composite score for perceived emotional support, with a higher score indicating greater emotional support. Participants with four or more missing items were classified as missing for emotional support. For those with fewer than four missing items, we imputed missing values using the mean of the available responses. The Cronbach's alpha for the emotional support scale in this sample was 0.89, indicating strong internal consistency. We standardized structural and functional social network characteristics to facilitate comparison.

#### 2.2.3 Cognitive function

Executive function and verbal episodic memory performance were assessed every 6 months, using the Spanish and English Neuropsychological Assessment Scales (SENAS).^25^ The SENAS, a battery of cognitive tests, has previously undergone extensive development for valid comparisons of cognitive change across groups with diverse racial, ethnic, and linguistic backgrounds.^25^ We focused on verbal episodic memory and executive function domains in the present analyses. While SENAS also assesses semantic memory, this domain was not included in the phone‐based interviews conducted during the pandemic and was therefore excluded from our analyses. Executive function composite scores were derived from performance on category fluency, phonemic (letter) fluency, and working memory tasks (Digit Span Backward, list sorting). Verbal episodic memory composite scores were based on a multi‐trial word‐list‐learning task that assesses participants’ ability to learn and recall verbal information through immediate and delayed free recall.^26^ Executive function and verbal episodic memory scores across all assessments were z‐standardized using the baseline sample mean and standard deviation.

#### 2.2.4 All‐cause mortality

All‐cause mortality was documented through multiple sources, including KPNC electronic medical records, the California State Mortality File, and Social Security Death records. Additionally, deaths were recorded when interviewers were notified of a participant's passing during attempts to schedule follow‐up interviews.

#### 2.2.5 Covariates

All covariates were assessed at baseline and included age (centered at the baseline sample mean), sex/gender obtained from electronic medical records (men [reference], women), self‐reported race and ethnicity (non‐Hispanic Asian, non‐Hispanic Black, Hispanic, or non‐Hispanic White [reference]), educational attainment (high school or lower [reference], some college but no college credential, associate degree, or bachelor's degree or more), and marital status (married [reference], never married, separated/divorced, widowed). Additionally, we adjusted for interview mode (in‐person [reference] vs. phone) to account for mode changes during the COVID‐19 pandemic.

### 2.3 Statistical analysis

We first summarized the characteristics of the sample overall, by sex/gender and loss‐to‐follow‐up status. We then fit separate models for executive function and verbal episodic memory, and for each social network characteristic to estimate the associations of social networks with cognitive performance, decline, and mortality using Bayesian joint models for longitudinal and survival data.^27^ These joint models fit two submodels: (1) a linear mixed‐effect model for repeated measures of cognitive function; and (2) a Cox proportional‐hazard model for time from baseline to death. The joint modeling approach links two models via shared random effects; simultaneous estimation of longitudinal and survival models allows the two models to borrow information from one another to improve estimation and account for loss to follow‐up due to death. For lost to follow‐up due to other reasons, the linear mixed‐effects models assume this information is missing at random, that is, the probability of missingness depends only on the observed data.

The linear mixed effects model considered cognitive function as the outcome with time since baseline (years) as the time scale. Both random intercepts and slopes were included. Each model included a single social network variable, covariates, interactions between covariates and time, and interactions between the social network variable and time. The Cox model for time to death accounted for cognitive function and covariates. The joint model parameters were estimated under a Bayesian framework using Markov chain Monte Carlo (MCMC) methods via the R package *JMbayes2*.^27^

Throughout the analyses, to account for potential practice effects (PE), we used a preliminary model‐based constrained practice effect approach. This approach estimated PE using pre‐pandemic data, which were then imposed across the full sample, including both pre‐ and intra‐pandemic observations. Further methodological details on the PE adjustment are provided elsewhere.^28^ Then, we refit all models stratified by sex/gender. The analyses were conducted using complete case analyses. While the characteristics between included versus excluded participants were largely similar, those who were excluded were, on average, older and had lower executive function and episodic memory scores at baseline compared with those included in the analyses (see Table S1). We also ran the primary analyses of the associations between social networks and cognitive function and mortality using imputed data. Specifically, we generated ten imputed datasets and pooled the estimates using Rubin's rules.[Table alz71600-tbl-0001]

#### 2.3.1 Exploratory and sensitivity analysis

Given that over half of the participants were widowed, we conducted an exploratory analysis restricted to widowed older adults to examine whether the associations of social network characteristics, cognitive function, and mortality differed in this subgroup. While the sample size within each race and ethnic group is small, we included stratified analyses by race and ethnicity as an exploratory analysis. These results should be interpreted with caution, but they may provide valuable information for future meta‐analyses.

We also conducted sensitivity analyses to assess the robustness of our findings. Since depressive symptoms may act as either a mediator or confounder, we fit models that additionally adjusted for depressive symptoms. Moreover, since social network size and composition may confound the associations of perceived emotional support with cognitive function and mortality, we fit a model that included all three social network characteristics to examine whether the association of emotional support with cognitive outcomes and mortality was independent of structural network characteristics. All analyses were performed using R version 4.2.1.

## 3 RESULTS

### 3.1 Demographic characteristics of the analytical sample

The mean age of our analytical sample (*n* = 677) was 93 years (standard deviation [SD] = 2.2; interquartile range [IQR] = 91.2–94.0), and 61.2% were women. The sample included 24.4% Asian participants, 24.5% Black participants, 19.8% Hispanic participants, and 31.3% White participants. About one‐third of the participants had attained a high school degree or less, and 60.3% were widowed at baseline.

Compared with men in the sample, women were more likely to be non‐White individuals, have no more than a high‐school education, and were much more likely to be widowed. They also reported greater emotional support, had more friend‐focused social networks, and had higher executive function and verbal episodic memory performance scores than men (see Table 1). Social network characteristics were moderately correlated, with correlation coefficients ranging from 0.24 to 0.41 (Figure S1).

**TABLE 1 alz71600-tbl-0001:** Baseline characteristics overall and by sex/gender in the LifeAfter90 Study.

	All	Women	Men
Parameter	*N* = 677	*N* = 414	*N* = 263
Age at baseline, mean (SD)	92.9 (2.2)	93.1 (2.3)	92.8 (2.2)
Sex/gender, *N* (%)			
Men	263 (38.8)	N/A	N/A
Women	414 (61.2)	N/A	N/A
Race and ethnicity, *N* (%)			
NH Asian	165 (24.4)	88 (21.3)	77 (29.3)
NH Black	166 (24.5)	109 (26.3)	57 (21.7)
Hispanic	134 (19.8)	92 (22.2)	42 (16.0)
NH White	212 (31.3)	125 (30.2)	87 (33.1)
Educational attainment, *N* (%)			
High school or less	232 (34.3)	161 (38.9)	71 (27.0)
Some college but no degree	134 (19.8)	88 (21.3)	46 (17.5)
Associate degree	69 (10.2)	42 (10.1)	27 (10.3)
Bachelor's degree	242 (35.7)	123 (29.7)	119 (45.2)
Marital status, *N* (%)			
Married	191 (28.2)	44 (10.6)	147 (55.9)
Never married	17 (2.5)	10 (2.4)	7 (2.7)
Separated/divorced	61 (9.0)	50 (12.1)	11 (4.2)
Widowed	408 (60.3)	310 (74.9)	98 (37.3)
Baseline social network size, mean (SD)	10.2 (8.6)	10.0 (9.1)	10.5 (7.8)
Baseline social network composition, mean (SD)	33.3 (24.7)	34.7 (24.4)	31.0 (25.0)
Baseline emotional support, mean (SD)	35.8 (9.1)	36.7 (9.1)	34.3 (9.0)
Baseline standardized executive function, mean (SD)	0 (1)	0.01 (1.04)	−0.02 (0.93)
Baseline standardized verbal episodic memory, mean (SD)	0 (1)	0.12 (1.04)	−0.18 (0.91)

*Note*: Percentages represent the proportion of individuals within each group who have the specified characteristic. Social network composition was calculated as the number of friends divided by the total number of individuals in a participant's social network*100, with higher scores indicating a greater proportion of friends and lower scores indicating a greater proportion of family members. The table presents raw social network scores, while standardized scores were used in the analysis.

Abbreviations: NH, non‐Hispanic; SD, standard deviation.

### 3.2 Characteristics of individuals who remained in the study versus those who were lost to follow‐up

During an average follow‐up period of 1.63 years (SD = 1.18, maximum: 3.72), 163 (24.1%) participants died, and 45 (6.6%) participants were lost to follow‐up for other reasons. Compared with individuals who remained in the study, those who died or were lost to follow‐up were more likely to be older, men, non‐Hispanic White individuals, and to have lower educational attainment at baseline. They also had smaller social networks, weaker emotional support, and had lower executive function and verbal episodic memory performance scores at baseline (see Table 2).

**TABLE 2:** Baseline characteristics in the overall population and by lost‐to‐follow‐up status in the LifeAfter90 Study.

	Deceased	Left the study	Remained in the study
Parameter	*N* = 163	*N* = 45	*N* = 469
Age at baseline, mean (SD)	93.6 (2.5)	93.0 (2.2)	92.7 (2.1)
Sex/gender, *N* (%)			
Men	70 (42.9)	18 (40.0)	175 (37.3)
Women	93 (57.1)	27 (60.0)	294 (62.7)
Race and ethnicity, *N* (%)			
NH Asian	30 (18.4)	16 (35.6)	119 (25.4)
NH Black	40 (24.5)	9 (20.0)	117 (24.9)
Hispanic	24 (14.7)	8 (17.8)	102 (21.7)
NH White	69 (42.3)	12 (26.7)	131 (27.9)
Educational attainment, *N* (%)			
High school or lower	64 (39.3)	21 (46.7)	147 (31.3)
Some college but no degree	30 (18.4)	9 (20.0)	95 (20.3)
Associate degree	13 (8.0)	5 (11.1)	51 (10.9)
Bachelor's degree	56 (34.4)	10 (22.2)	176 (37.5)
Marital status, *N* (%)			
Married	51 (31.3)	11 (24.4)	129 (27.5)
Never married	4 (2.5)	0 (0)	13 (2.8)
Separated/divorced	15 (9.2)	2 (4.4)	44 (9.4)
Widowed	93 (57.1)	32 (71.1)	283 (60.3)
Baseline social network size, mean (SD)	9.1 (6.4)	9.3 (7.7)	10.6 (9.3)
Baseline social network composition, mean (SD)	30.6 (24.9)	31.0 (20.9)	34.4 (24.9)
Baseline emotional support, mean (SD)	35.1 (9.3)	32.4 (9.2)	36.4 (9.0)
Baseline executive function, mean (SD)	−0.4 (1.0)	−0.4 (0.8)	−0.03 (1.0)
Baseline verbal episodic memory, mean (SD)	−0.5 (0.8)	−0.5 (0.9)	−0.1 (1.0)

*Note*: Percentages represent the proportion of individuals within each group who have the specified characteristic. Social network composition was calculated as the number of friends divided by the total number of individuals in a participant's social network*100, with higher scores indicating a greater proportion of friends and lower scores indicating a greater proportion of family members. The table presents raw social network scores, while standardized scores were used in the analysis.

Abbreviations: NH, non‐Hispanic; SD, standard deviation.

### 3.3 Associations of structural and functional characteristics of social networks with cognitive function levels

**FIGURE 1:**
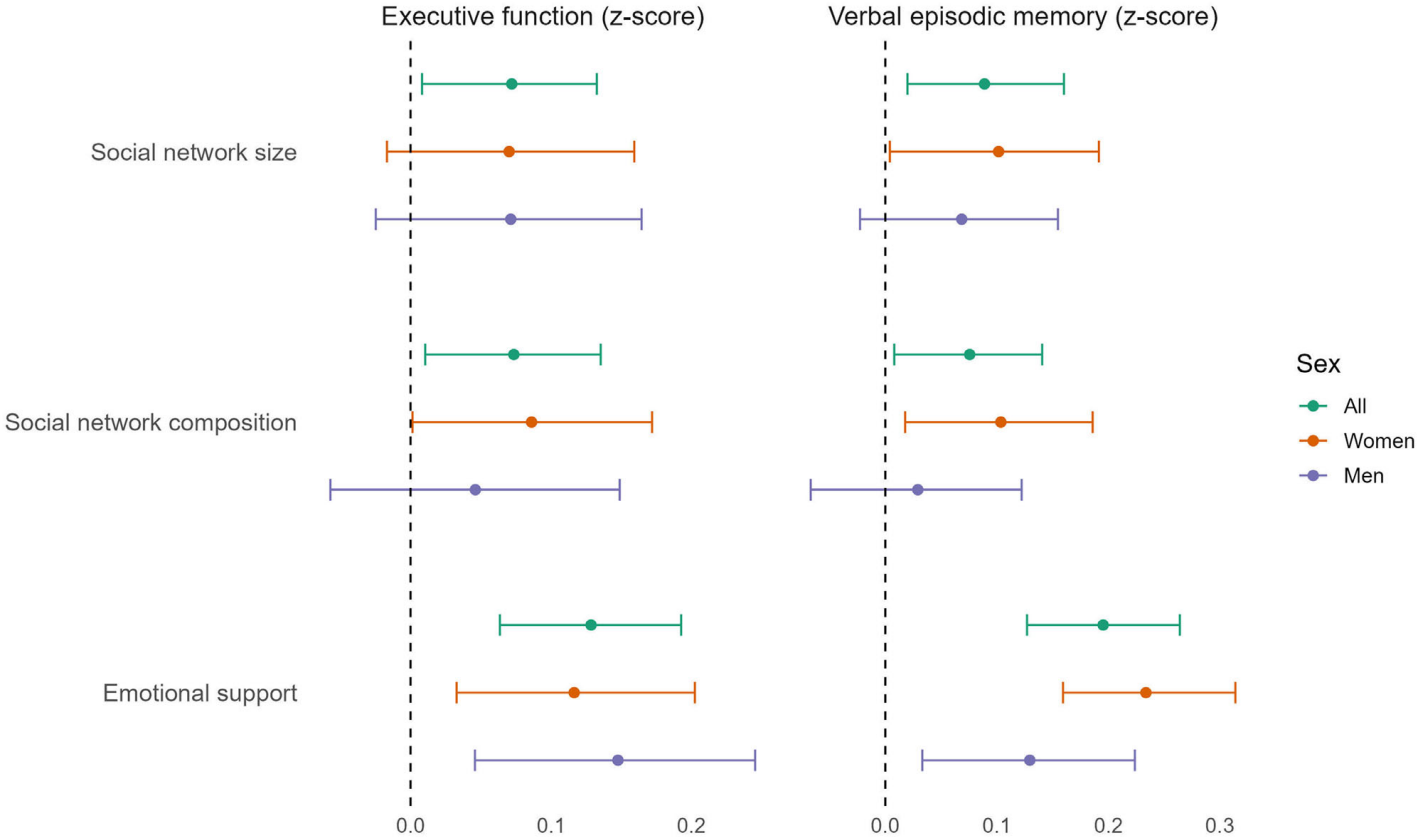
Estimated associations of structural and functional social networks with levels of cognitive function at baseline in the LifeAfter90 Study. Lines represent 95% confidence intervals. Model was adjusted for age, interview mode, sex/gender, race and ethnicity, education, and marital status. Both the social network and the cognitive function scores were standardized to baseline mean and SD. SD, standard deviation.

Each one‐unit greater in social network size at baseline was associated with 0.07 SD higher average executive function score (95% confidence interval [CI] = 0.01, 0.13) and 0.09 SD higher average verbal episodic memory score (95% CI = 0.02, 0.16). Having more friends (vs. family) in one's network was associated with better executive function ($\hat{\beta }$ = 0.07, 95% CI = 0.01, 0.14) and verbal episodic memory z‐scores ($\hat{\beta }$ = 0.08, 95% CI = 0.01, 0.14) (see Figure 1). Greater emotional support was associated with better performance in executive function ($\hat{\beta }$ = 0.13, 95% CI = 0.06, 0.19) and verbal episodic memory ($\hat{\beta }$ = 0.20, 95% CI = 0.13, 0.26). Results from analyses using imputed data remained consistent with those observed in the complete case analyses (Figure S2).

### 3.4 Associations of structural and functional characteristics of social networks with decline in cognitive function

We found no evidence of associations between either structural or functional social networks and the annual rate of decline in executive function or verbal episodic memory performance. For example, the estimated association of social network size with the annual rate of decline in executive function was ${\hat{\beta }}_{\mathrm{social}\;\mathrm{network}\;\mathrm{size}*\mathrm{time}\;}$= −0.01, 95% CI = −0.04, 0.01. Similarly, none of the social network characteristics were associated with mortality (e.g., the estimated association between social network size and mortality hazard is ${\hat{HR}}_{\mathrm{social}\;\mathrm{network}\;\mathrm{size}\;}$= 0.90, 95% CI = 0.69, 1.12; see Table 3). Results were consistent when using imputed data (Table S2).

**TABLE 3:** Estimated joint model coefficients for structural and functional social networks with executive function, verbal episodic memory, and mortality from the LifeAfter90 Study (*N* = 677).

			All	Women	Men
Independent variable	Model	Dependent variable	Estimate (95% CI)	Estimate (95% CI)	Estimate (95% CI)
Social network size *Time	Longitudinal	Executive Function	−0.01 (−0.04, 0.01)	−0.02 (−0.05, 0.01)	−0.01 (−0.05, 0.04)
Social Network Size	Cox	Mortality	0.90 (0.69, 1.12)	0.83 (0.55, 1.13)	0.96 (0.60, 1.40)
Social network size *Time	Longitudinal	Verbal episodic memory	0.00 (−0.04, 0.03)	0.00 (−0.05, 0.06)	0.02 (−0.03, 0.08)
Social Network Size	Cox	Mortality	0.91 (0.70, 1.14)	0.84 (0.58, 1.14)	0.99 (0.64, 1.36)
Social network composition*Time	Longitudinal	Executive Function	0.00 (−0.03, 0.02)	−0.01 (−0.04, 0.02)	0.00 (−0.05, 0.04)
Social network composition	Cox	Mortality	0.91 (0.73, 1.11)	0.88 (0.65, 1.15)	0.87 (0.61, 1.25)
Social network composition*Time	Longitudinal	Verbal episodic memory	0.01 (−0.01, 0.04)	0.00 (−0.03, 0.04)	0.02 (−0.03, 0.08)
Social network composition	Cox	Mortality	0.92 (0.74, 1.12)	0.87 (0.63, 1.16)	0.88 (0.61, 1.30)
Emotional support *Time	Longitudinal	Executive Function	0.00 (−0.03, 0.03)	−0.01 (−0.04, 0.02)	0.02 (−0.04, 0.07)
Emotional support	Cox	Mortality	1.05 (0.85, 1.27)	1.18 (0.86, 1.59)	0.92 (0.64, 1.33)
Emotional support *Time	Longitudinal	Verbal episodic memory	−0.01 (−0.04, 0.02)	−0.01 (−0.04, 0.02)	−0.02 (−0.09, 0.03)
Emotional support	Cox	Mortality	1.06 (0.83, 1.31)	1.19 (0.86, 1.63)	0.90 (0.65, 1.29)

*Note*: Linear mixed effect models were adjusted for age, interview mode, sex/gender, race and ethnicity, education levels, and marital status. The executive function and verbal episodic memory scores were standardized to baseline mean and SD.

Abbreviations: CI, confidence interval; SD, standard deviation.

### 3.5 Sex‐/gender‐stratified findings

In sex‐/gender‐stratified analyses, the associations of structural and functional social network characteristics with baseline executive function and episodic memory were generally consistent across groups, though estimates were less precise among men than women (Figure 1). No associations were observed between social network characteristics and the rate of cognitive decline in either men or women. Similarly, no associations were found between social networks and mortality risks in either group (Table 3).

### 3.6 Exploratory and sensitivity analysis results

In an exploratory analysis restricted to widowed older adults, the associations were largely consistent with those observed in the full sample. Specifically, both emotional support and social network size were associated with higher baseline executive function and verbal episodic memory (Figure S3), but no associations were found with the rate of decline in cognitive function or mortality (Table S3). After stratifying by race and ethnicity, the direction of associations was generally consistent, except for having a higher proportion of friends versus family among Asian American older adults, which showed a negative association with baseline executive function. Many estimates crossed the null, likely due to small sample sizes (Figure S4). Higher social network size was associated with slower executive function decline among Asian American older adults, while greater emotional support was linked to faster decline among Black American older adults (Table S4).

After additional adjustment for social network size and composition, emotional support remained associated with baseline levels of executive function and verbal episodic memory, but not with rates of decline or mortality. When models were additionally adjusted for baseline depressive symptoms, social network composition was no longer associated with either executive function or verbal episodic memory in the full sample (Figure S5). However, greater emotional support was associated with a faster rate of cognitive decline. Among women, higher emotional support was linked to a greater hazard of mortality, while larger social network size and a higher proportion of friends (vs. family) in the network were associated with a slower decline in verbal episodic memory (Table S5).

## 4 DISCUSSION

This study fit joint models to estimate the associations of social networks with cognitive function, decline, and all‐cause mortality and to explore potential sex/gender differences in these associations among a racially and ethnically diverse cohort of older adults aged 90 years and older. Both structural and functional social network characteristics were associated with higher levels of cognitive function, with similar patterns observed for men and women. However, neither structural nor functional social network characteristics were associated with short‐term cognitive decline or mortality, either in the overall sample or across sex/gender subgroups.

Our findings that social networks are associated with greater cognitive function levels are consistent with most previous research conducted in younger older adult populations.^8^, ^10^ Although these results should be interpreted cautiously due to the potential for reverse causation, our study adds further evidence that the cognitive benefits of social networks persist into very old age. Moreover, we found that friend‐focused (vs. family‐focused) social networks were associated with better cognitive function. This finding supports the growing body of evidence that suggests friendships protect cognitive health.^23^, ^29^, ^30^, ^31^ Compared with family‐focused networks, friend‐focused social networks may offer more opportunities for engaging in social and leisure activities, which have been linked to better cognitive function.^32^ It has also been suggested that, as age increases, older adults may maintain high‐quality friendships while letting go of low‐quality relationships to optimize well‐being.^23^, ^33^ In contrast, disconnecting from unsatisfactory family relationships may be more challenging, and negative family interactions (e.g., conflict) can introduce undue stress that is harmful to cognitive functioning.^34^ Reverse causation may also contribute to these observed associations, as individuals with poorer cognitive function may increasingly rely on family members for social support, thereby increasing the presence of family members in their network.^35^ Notably, these associations were attenuated to null after further adjustment for depressive symptoms, suggesting that depressive symptoms may partially account for the observed relationships.

Our findings highlight the importance of considering both structural and functional components of social networks in our understanding of cognitive function. Compared with structural characteristics of social networks, functional aspects (i.e., emotional support) were more strongly associated with cognitive function. These associations were largely consistent across sex/gender and racial and ethnic groups and were robust in sensitivity analyses adjusting for depressive symptoms or including the other two social network characteristics in the model. The observed associations between perceived emotional support and cognitive function suggest that social networks may influence cognitive function through stress‐buffering mechanisms among older adults in advanced old age; however, this hypothesis warrants further investigation. While previous research has suggested that the associations of social networks with cognitive function vary across cognitive domains, we found no such evidence. Instead, structural and functional social networks showed similar associations with both executive function and verbal episodic memory levels. This may indicate that social network characteristics influence cognitive function through mechanisms that affect both executive function and verbal episodic memory, such as systemic inflammation or vascular risk factors. However, further research is needed to test these potential pathways.

We found little evidence that structural or functional social network characteristics were associated with cognitive decline or all‐cause mortality over an average follow‐up of 1.63 years. Although this follow‐up period is relatively short, it represents a meaningful proportion of the remaining life expectancy for adults aged 90 years and older. Even over this short period, nearly 24% of participants died. While our findings do not support previous studies that have suggested the protective effects of social networks on cognitive decline and mortality,^10^, ^36^ wide confidence intervals indicate that more evidence is needed. The associations with cognitive levels help approximate how much of a difference in rate of cognitive change is plausible. For example, a 0.07 SD difference in executive function per unit difference in social network size may reflect the cumulative impact of approximately 20 years of cognitive aging in this population of adults aged 90 and older. If so, we would expect the effect of social networks on the annual rate of cognitive decline to be roughly one‐twentieth the size of the association with baseline level. Our estimates were not sufficiently precise to detect such a small effect.

Greater emotional support was associated with faster cognitive decline among Black older adults and with higher mortality among women. Several factors may explain these unexpected findings. For example, individuals who experience early symptoms of cognitive decline may receive more emotional support from close network members. Therefore, reverse causation may account for part of the observed associations. Additionally, receiving support may lead to reciprocal emotional labor.^37^ Women disproportionately provide care to others, and they may experience stress from these reciprocal obligations, which could contribute to worse health outcomes. Finally, selective survival may also play a role. Individuals with weaker social networks who experience fast cognitive decline might have already died before age 90. By contrast, those who survive to age 90 with limited social support may be particularly resilient, which could obscure a protective effect of emotional support on health outcomes.

This study has several limitations. As with many other studies on late‐life risk factors for cognitive health, we cannot rule out the possibility of reverse causation between social network characteristics and cognitive function. For example, individuals with better cognitive function may be more likely to maintain friendships, whereas those with poorer cognitive function may withdraw from social interactions or activities. Our study was also restricted to long‐term members of a large, integrated healthcare system, which may limit the generalizability of our findings, particularly to populations with limited access to healthcare. Nevertheless, given the scarcity of research on social networks and cognitive function among older adults in advanced old age, our findings are important additions to the literature. Our social network measures were based on self‐report and may be prone to recall bias or social desirability bias. Although we examined both structural and functional aspects of social networks, we were unable to assess other relevant dimensions, such as frequency of contact, which were not measured in the LA90 Study. Residual confounding may also be present, as we did not have access to data on certain potential confounders, such as chronic health conditions. Although the inclusion of racially and ethnically diverse participants is a notable strength, race‐stratified analyses were considered exploratory due to the small size of each subgroup. However, as enrollment in the LA90 Study continues, the growing sample will allow for more rigorous disaggregation by race and ethnicity in future analyses. Lastly, we were unable to assess differences among sexual and gender minority participants. Future research should explore whether the associations between social networks, cognitive outcomes, and mortality vary across older adults with diverse sexual and gender identities.

Despite the limitations, our study has several strengths and innovations. A major strength is the focus on adults aged 90 years and older with diverse racial and ethnic identities. As noted, prior studies rarely include individuals in advanced old age, and nearly all such populations identify as non‐Hispanic White. Our study fills this critical knowledge gap and offers empirical evidence about the extent to which late‐life social networks influence cognitive function and mortality in a diverse population aged 90 years and older. Our inclusion of both structural and functional aspects of social networks, as well as different cognitive domains, allowed for a comprehensive assessment of the complex relationships between social networks, cognitive outcomes, and mortality. Additionally, while prior research has examined the link between social networks and cognition in older adults, few studies have formally addressed the impact of attrition due to death, a critical source of bias in very old populations.^23^, ^38^ Our joint modeling approach allowed us to account for attrition due to death while simultaneously estimating associations of social networks with both longitudinal and time‐to‐event outcomes.

## 5 CONCLUSION

In this racially and ethnically diverse cohort of adults aged 90 years and older, both late‐life functional and structural characteristics of social networks were associated with cognitive function, with generally consistent patterns observed across men and women. However, we found little evidence that social networks were associated with cognitive decline or mortality over the study period. This study contributes to the growing evidence on the social determinants of cognitive aging and longevity in older adults in advanced old age, a growing but understudied population. It is critical to develop targeted research and interventions for this distinct age group.

## CONFLICT OF INTEREST STATEMENT

The authors declare no conflicts of interests. Author disclosures are available in the Supporting Information.

## CONSENT STATEMENT

All participants provided written informed consent.

## Supporting information




Supporting Information

Supporting Information
