# Supplementary material for: Social network characteristics and cognitive function, decline, and mortality: A joint modeling approach
Source: Alzheimers Dement. 2026 Jul 12;22(7):e71600. doi: 10.1002/alz.71600 (PMC13357703; doi:10.1002/alz.71600)
Supplement: Supplementary file 2 — Supporting Information [file ALZ-22-e71600-s001.docx]

**Supplemental Document**

**Supplemental Figure 1**. Correlations among social network characteristics, cognitive outcomes, and mortality

**Supplemental Table 1.**  Characteristics of included (n =677) vs. excluded (n=80) participants

**Supplemental Figure 2.** Estimated associations of structural and functional social networks with levels of cognitive function at baseline in the Lifeafter90 Study (imputed data).

**Supplemental Table 2.** Estimated joint model coefficients for structural and functional social networks with executive function, verbal episodic memory, and mortality from the Lifeafter90 study (imputed data).

**Supplemental Figure 3.** Estimated associations of structural and functional social networks with levels of cognitive function at baseline in the Lifeafter90 Study among widowed older adults.

**Supplemental Table 3.** Estimated Joint Model Coefficients for Structural and Functional Social Networks with Executive Function, Verbal Episodic Memory, and Mortality from the LifeAfter90 Study among widowed older adults.

**Supplemental Figure 4.** Estimated associations of structural and functional social networks with levels of cognitive function at baseline in the Lifeafter90 Study, stratified by race and ethnicity status.

**Supplemental Table 4.** Estimated joint model coefficients for structural and functional social networks with executive function, verbal episodic memory, and mortality from the Lifeafter90 study, stratified by race and ethnicity status.

**Supplemental Figure 5.** Estimated associations of structural and functional social networks with levels of cognitive function at baseline in the Lifeafter90 Study, additionally adjusted for depressive symptoms at baseline.

**Supplemental Table 5.** Estimated joint model coefficients for structural and functional social networks with executive function, verbal episodic memory, and mortality from the Lifeafter90 study, additionally adjusted for depressive symptoms.

**Supplemental Figure 1**. Correlations among social network characteristics, cognitive outcomes, and mortality


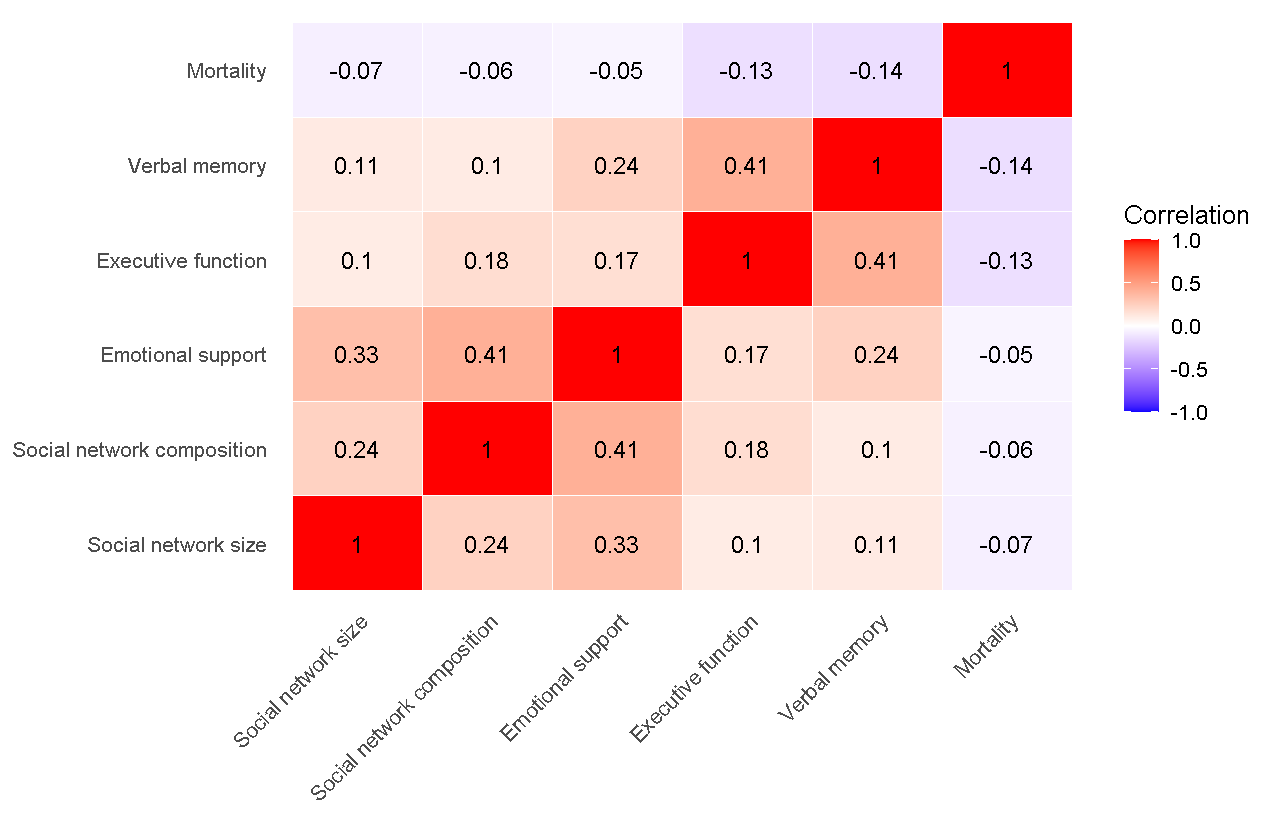


Note: Mortality is a binary variable (coded as 0 = alive, 1 = deceased). Point-biserial correlations were computed between mortality and other variables. These correlations should be interpreted with caution and are presented for descriptive purposes only.

**Supplemental Table 1**. Characteristics of the included (N=677) vs. excluded (N=80) participants.

|  | Included (N=677) | Excluded (N=80) |
| --- | --- | --- |
| **Age, Mean (SD)** | 92.94 (2.24) | 93.70 (3.11) |
| **Female, N (%)** | 414 (61.2) | 46 (57.5) |
| **Race and ethnicity, N (%)** |  |  |
| NH White | 212 (31.3) | 21 (26.2) |
| Latinx | 134 (19.8) | 18 (22.5) |
| NH Black | 166 (24.5) | 17 (21.2) |
| NH Asian | 165 (24.4) | 24 (30.0) |
| **Education, N (%)** |  |  |
| High school or less | 232 (34.3) | 27 (33.8) |
| Some college | 134 (19.8) | 15 (18.8) |
| Bachelor's degree | 242 (35.7) | 33 (41.2) |
| Associate degree | 69 (10.2) | 5 (6.2) |
| **Marital status, N (%)** |  |  |
| Married | 180 (26.6) | 23 (28.7) |
| Never married | 16 (2.4) | 0 (0.0) |
| Separated/Divorced | 57 (8.4) | 7 (8.8) |
| Widowed | 397 (58.6) | 50 (62.5) |
| **Social network size, Mean (SD)** | 10.19 (8.58) | 8.38 (5.45) |
| **Social network composition, Mean (SD)** | 33.26 (24.67) | 27.19 (26.20) |
| **Emotional support, Mean (SD)** | 35.80 (9.11) | 34.02 (11.21) |
| **Executive function, Mean (SD)** | -0.46 (0.66) | -0.76 (0.67) |
| **Verbal memory, Mean (SD)** | -0.60 (0.80) | -0.88 (0.92) |

**Note.** SD = Standard deviation; NH = non-Hispanic. Percentages represent the proportion of individuals within each group who have the specified characteristic. Social network composition was calculated as the number of friends divided by the total number of individuals in a participant’s social network*100, with higher scores indicating a greater proportion of friends and lower scores indicating a greater proportion of family members. The table presents raw social network scores, while standardized scores were used in the analysis.


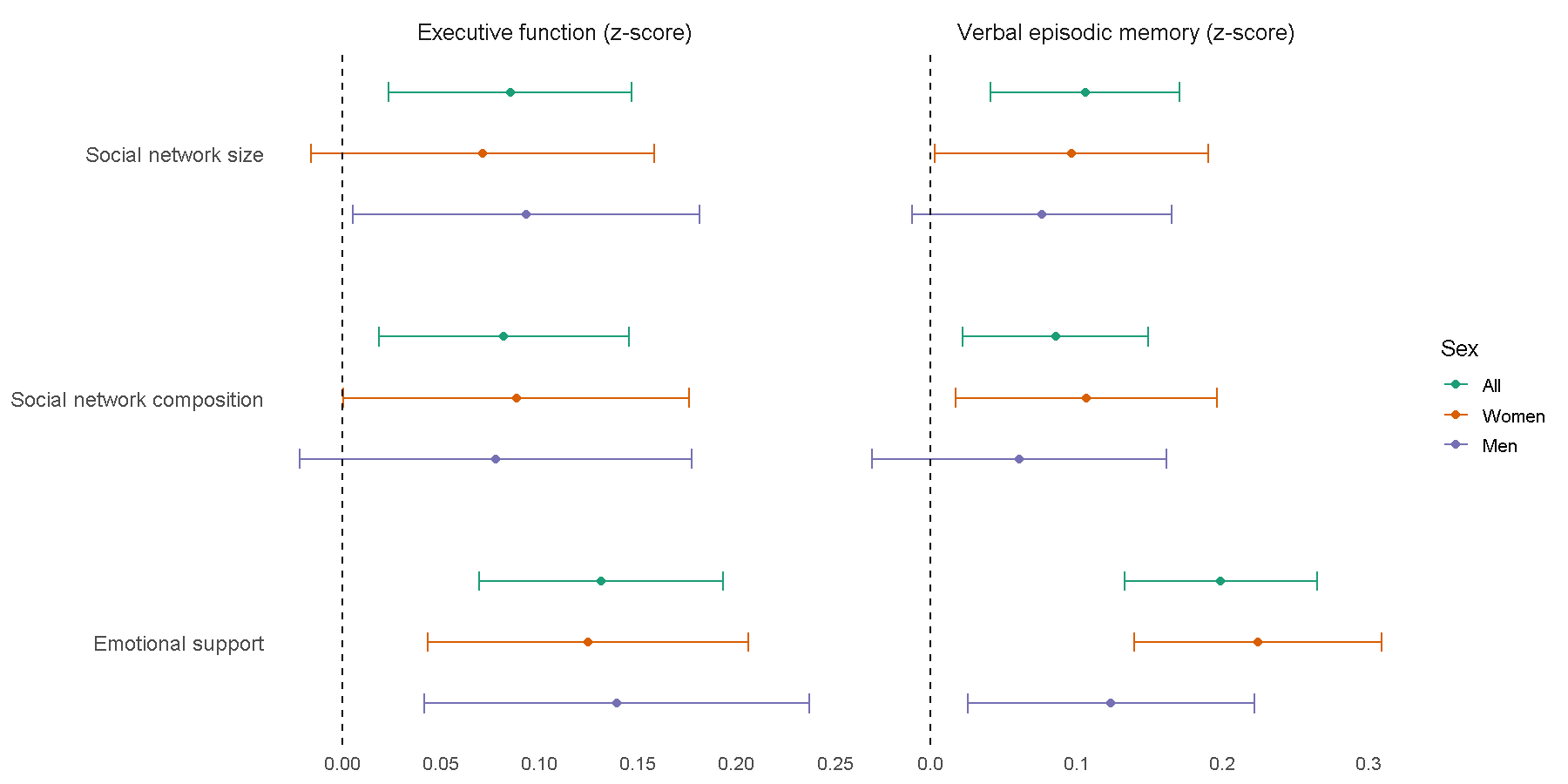


**Supplemental Figure 2.** Estimated associations of structural and functional social networks with levels of cognitive function at baseline in the Lifeafter90 Study (imputed data). Lines represent 95% confidence intervals. *Note.* Model was adjusted for age, interview mode, sex/gender, race and ethnicity, education, and marital status. Both the social network and the cognitive function scores were standardized to baseline mean and SD.

**Supplemental Table 2.** Estimated joint model coefficients for structural and functional social networks with executive function, verbal episodic memory, and mortality from the Lifeafter90 study (imputed data).

| **Independent Variable** | **Model** | **Dependent Variable** | **All** | **Women** | **Men** |
| --- | --- | --- | --- | --- | --- |
| Social network size * Time | Longitudinal | Executive Function | -0.02 (-0.04, 0.01) | -0.02 (-0.05, -0.01) | -0.01 (-0.05, -0.03) |
| Social Network Size | Cox | Mortality | 0.92 (0.73, 1.15) | 0.87 (0.64, 1.18) | 0.94 (0.67, 1.32) |
|  |  |  |  |  |  |
| Social network size * Time | Longitudinal | Verbal Episodic Memory | -0.01 (-0.04, 0.01) | 0.00 (-0.04, 0.04) | 0.01 (-0.02, 0.05) |
| Social Network Size | Cox | Mortality | 0.92 (0.74, 1.16) | 0.87 (0.65, 1.19) | 0.98 (0.70, 1.38) |
|  |  |  |  |  |  |
| Social network composition * Time | Longitudinal | Executive Function | 0.00 (-0.03, 0.02) | -0.01 (-0.05, 0.02) | -0.01 (-0.05, 0.03) |
| Social Network Composition | Cox | Mortality | 0.91 (0.74, 1.12) | 0.88 (0.68, 1.14) | 0.87 (0.63, 1.20) |
|  |  |  |  |  |  |
| Social network composition * Time | Longitudinal | Verbal Episodic Memory | 0.02 (-0.01, 0.04) | 0.00 (-0.04, 0.04) | 0.02 (-0.02, 0.06) |
| Social Network Composition | Cox | Mortality | 0.92 (0.75, 1.13) | 0.89 (0.68, 1.15) | 0.90 (0.64, 1.27) |
|  |  |  |  |  |  |
| Emotional support * Time | Longitudinal | Executive Function | 0.00 (-0.03, 0.02) | -0.01 (-0.04, 0.03) | 0.01 (-0.03, 0.05) |
| Emotional Support | Cox | Mortality | 1.05 (0.85, 1.30) | 1.17 (0.89, 1.55) | 0.94 (0.68, 1.31) |
|  |  |  |  |  |  |
| Emotional support * Time | Longitudinal | Verbal Episodic Memory | -0.02 (-0.05, 0.01) | 0.01 (-0.03, 0.05) | -0.01 (-0.05, 0.02) |
| Emotional Support | Cox | Mortality | 1.06 (0.86, 1.31) | 1.20 (0.90, 1.60) | 0.92 (0.67, 1.28) |

*Note.* Linear mixed effect models were adjusted for age, interview mode, sex/gender, race and ethnicity, education levels, and marital status. The Executive function and verbal episodic memory scores were standardized to baseline mean and SD.


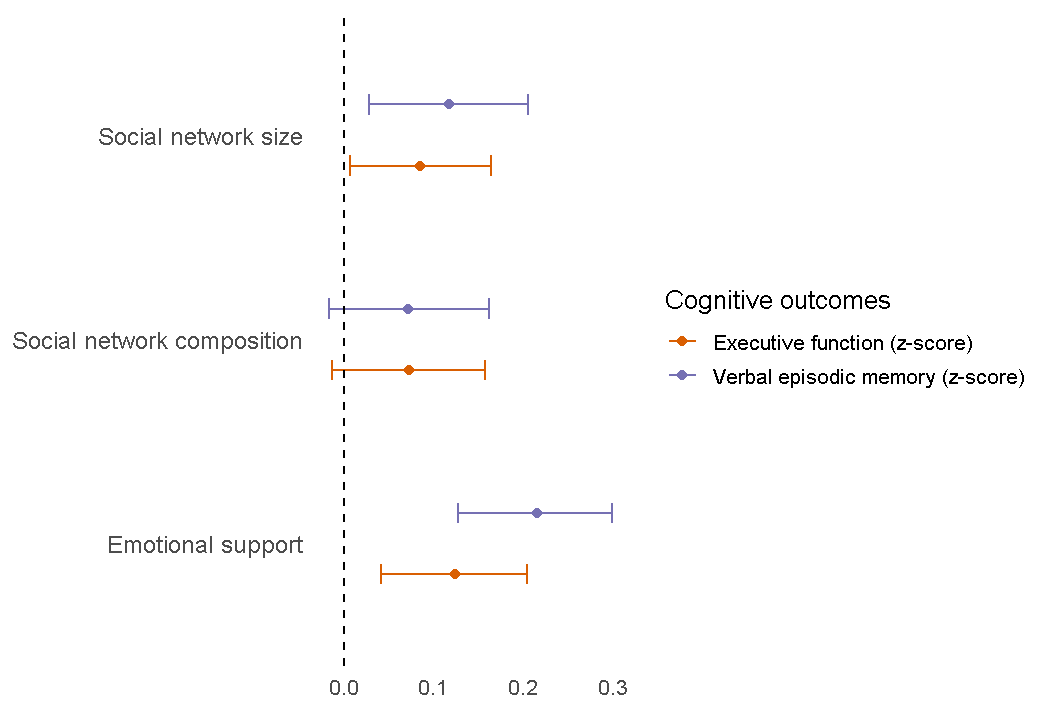


**Supplemental Figure 3.** Estimated associations of structural and functional social networks with levels of cognitive function at baseline in the Lifeafter90 Study among widowed older adults. Lines represent 95% confidence intervals. *Note.* Model was adjusted for age, interview mode, sex/gender, race and ethnicity, education, marital status, and depressive symptoms. Both the social network and the cognitive function scores were standardized to baseline mean and SD.

**Supplemental Table 3.** Estimated joint model coefficients for structural and functional social networks with executive function, verbal episodic memory, and mortality from the Lifeafter90 study among widowed older adults.

| **Independent Variable** | **Model** | **Dependent Variable** | **Widowed older adults** |
| --- | --- | --- | --- |
|  |  |  | **Estimate (95% CI)** |
| Social network size * Time | Longitudinal | Executive Function | -0.02 (-0.06, 0.01) |
| Social Network Size | Cox | Mortality | 0.97 (0.69, 1.30) |
| Social network size * Time | Longitudinal | Verbal Episodic Memory | -0.02 (-0.06, 0.01) |
| Social Network Size | Cox | Mortality | 0.97 (0.70, 1.30) |
| Social network composition * Time | Longitudinal | Executive Function | -0.02 (-0.05, 0.02) |
| Social Network Composition | Cox | Mortality | 0.89 (0.68, 1.19) |
| Social network composition * Time | Longitudinal | Verbal Episodic Memory | -0.01 (-0.05, 0.03) |
| Social Network Composition | Cox | Mortality | 0.89 (0.68, 1.18) |
| Emotional support * Time | Longitudinal | Executive Function | 0.01 (-0.03, 0.05) |
| Emotional Support | Cox | Mortality | (0.77, 1.35) |
| Emotional support * Time | Longitudinal | Verbal Episodic Memory | 0.01 (-0.04, 0.05) |
| Emotional Support | Cox | Mortality | 1.05 (0.80, 1.42) |

*Note.* Linear mixed effect models were adjusted for age, interview mode, sex/gender, race and ethnicity, education levels, marital status, and depressive symptoms. The Executive function and verbal episodic memory scores were standardized to baseline mean and SD.


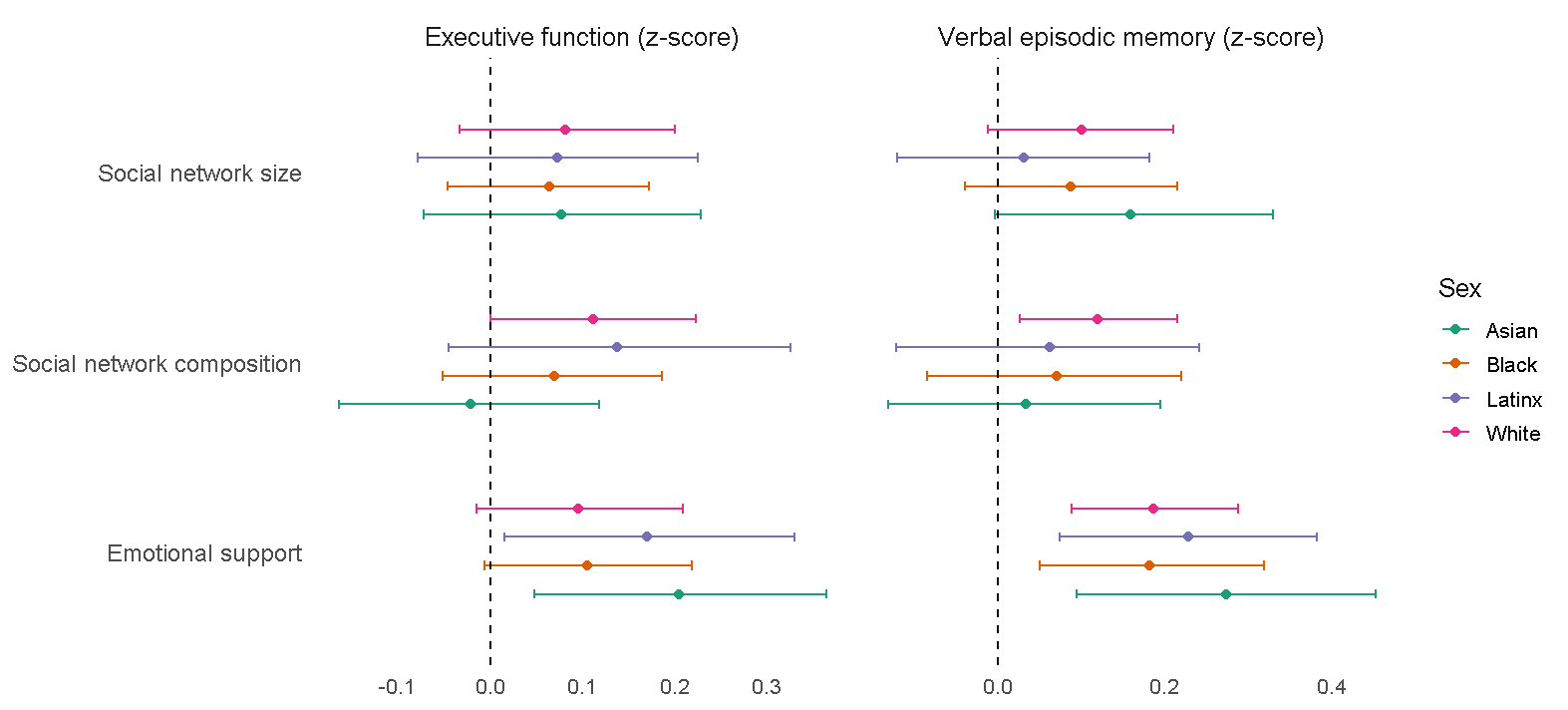


**Supplemental Figure 4.** Estimated associations of structural and functional social networks with levels of cognitive function at baseline in the Lifeafter90 Study, stratified by race and ethnicity. Lines represent 95% confidence intervals. *Note.* Model was adjusted for age, interview mode, sex/gender, race and ethnicity, education, marital status, and depressive symptoms. Both the social network and the cognitive function scores were standardized to baseline mean and SD.

**Supplemental Table 4.** Estimated joint model coefficients for structural and functional social networks with executive function, verbal episodic memory, and mortality from the Lifeafter90 study, stratified by race and ethnicity.

| **Independent Variable** | **Model** | **Dependent Variable** | **Non-Hispanic Asian**  **(n=165)** | **Non-Hispanic Black**  **(n=166)** | **Latinx**  **(n=134)** | **Non-Hispanic White**  **(n=234)** |
| --- | --- | --- | --- | --- | --- | --- |
| Social network size * Time | Longitudinal | Executive Function | -0.03 (-0.09, 0.04) | -0.02 (-0.06, 0.02) | 0.02 (-0.03, 0.08) | 0.00 (-0.03, 0.05) |
| Social Network Size | Cox | Mortality | 0.63 (0.26, 1.45) | 0.88 (0.61, 1.20) | 1.01 (0.68, 1.68) | 1.02 (0.70, 1.46) |
|  |  |  |  |  |  |  |
| Social network size * Time | Longitudinal | Verbal Episodic Memory | **0.05 (0.01, 0.09)** | -0.05 (-0.11, 0.00) | 0.01 (-0.06, 0.11) | 0.03 (-0.02, 0.06) |
| Social Network Size | Cox | Mortality | 0.67 (0.25, 1.47) | 0.95 (0.56, 1.52) | 0.8 (0.36, 1.45) | 0.91 (0.65, 1.27) |
|  |  |  |  |  |  |  |
| Social network composition * Time | Longitudinal | Executive Function | -0.01 (-0.07, 0.04) | 0.01 (-0.02, 0.04) | 0.00 (-0.07, 0.07) | -0.01 (-0.05, 0.03) |
| Social Network Composition | Cox | Mortality | 0.81 (0.42, 1.43) | 0.72 (0.44, 1.12) | 0.71 (0.37, 1.34) | 0.98 (0.72, 1.37) |
|  |  |  |  |  |  |  |
|  |  |  |  |  |  |  |
| Social network composition * Time | Longitudinal | Verbal Episodic Memory | -0.01 (-0.07, 0.07) | -0.01 (-0.08, 0.08) | 0.00 (-0.09, 0.09) | -0.01 (-0.04, 0.03) |
| Social Network Composition | Cox | Mortality | 0.84 (0.45, 1.48) | 0.72 (0.43, 1.15) | 0.67 (0.31, 1.39) | 1.06 (0.77, 1.44) |
|  |  |  |  |  |  |  |
| Emotional support * Time | Longitudinal | Executive Function | -0.05 (-0.11, 0.03) | **-0.04 (-0.06, -0.02)** | 0.01 (-0.04, 0.07) | 0.00 (-0.05, 0.05) |
| Emotional Support | Cox | Mortality | 0.98 (0.50, 1.88) | 0.97 (0.64, 1.47) | 1.38 (0.79, 2.54) | 1.07 (0.77, 1.49) |
|  |  |  |  |  |  |  |
| Emotional support * Time | Longitudinal | Verbal Episodic Memory | 0.03 (-0.05, 0.11) | -0.08 (-0.15, 0.00) | -0.01 (-0.12, 0.08) | -0.02 (-0.06, 0.02) |
| Emotional Support | Cox | Mortality | 1.02 (0.52, 1.98) | 1.01 (0.60, 1.78) | 1.32 (0.71, 2.55) | 1.13 (0.82, 1.60) |

*Note.* Linear mixed effect models were adjusted for age, interview mode, sex/gender, race and ethnicity, education levels, marital status, and depressive symptoms. The Executive function and verbal episodic memory scores were standardized to baseline mean and SD. Bolded estimates indicate *p* < 0.05.


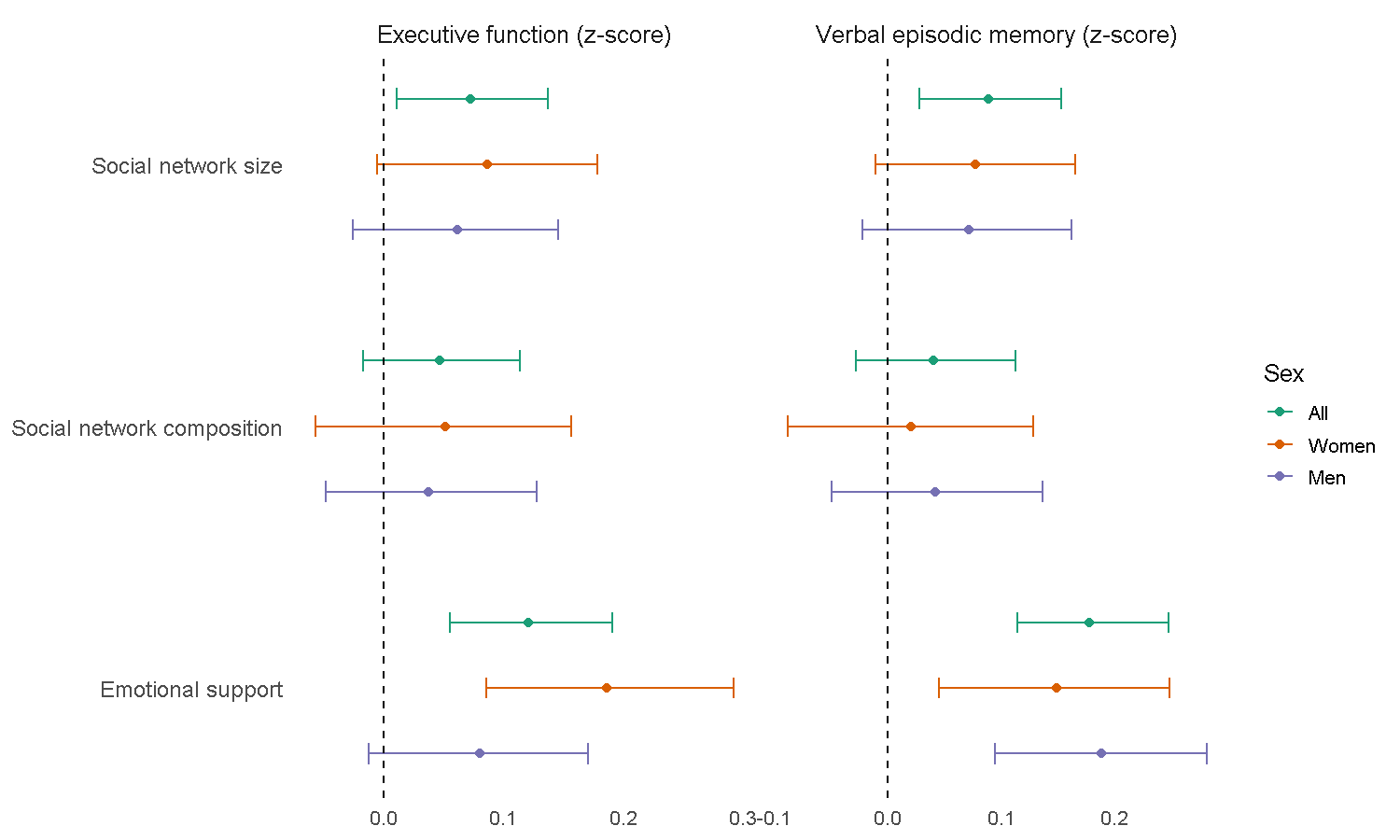


**Supplemental Figure 5.** Estimated associations of structural and functional social networks with levels of cognitive function at baseline in the Lifeafter90 Study, additionally adjusted for depressive symptoms at baseline. Lines represent 95% confidence intervals. *Note.* Model was adjusted for age, interview mode, sex/gender, race and ethnicity, education, marital status, and depressive symptoms. Both the social network and the cognitive function scores were standardized to baseline mean and SD.

**Supplemental Table 5.** Estimated joint model coefficients for structural and functional social networks with executive function, verbal episodic memory, and mortality from the Lifeafter90 study, additionally adjusted for depressive symptoms.

| **Independent Variable** | **Model** | **Dependent Variable** | **All** | **Women** | **Men** |
| --- | --- | --- | --- | --- | --- |
| Social network size * Time | Longitudinal | Executive Function | -0.01 (-0.04, 0.01) | -0.02 (-0.05, 0.02) | 0.00 (-0.04, 0.05) |
| Social Network Size | Cox | Mortality | 0.96 (0.74, 1.21) | 0.88 (0.61, 1.20) | 1.03 (0.70, 1.47) |
|  |  |  |  |  |  |
| Social network size * Time | Longitudinal | Verbal Episodic Memory | 0.00 (-0.03, 0.02) | **0.01 (0.00, 0.01)** | 0.03 (-0.02, 0.07) |
| Social Network Size | Cox | Mortality | 0.96 (0.75, 1.20) | 0.87 (0.58, 1.21) | 1.10 (0.75, 1.66) |
|  |  |  |  |  |  |
| Social network composition * Time | Longitudinal | Executive Function | 0.00 (-0.03, 0.02) | -0.01 (-0.04, 0.03) | 0.00 (-0.04, 0.03) |
| Social Network Composition | Cox | Mortality | 0.90 (0.72, 1.12) | 0.87 (0.65, 1.18) | 0.86 (0.61, 1.23) |
|  |  |  |  |  |  |
| Social network composition * Time | Longitudinal | Verbal Episodic Memory | 0.02 (-0.01, 0.04) | **0.02 (0.00, 0.03)** | 0.02 (-0.04, 0.06) |
| Social Network Composition | Cox | Mortality | 0.91 (0.72, 1.13) | 0.88 (0.64, 1.18) | 0.93 (0.65, 1.38) |
|  |  |  |  |  |  |
| Emotional support * Time | Longitudinal | Executive Function | -0.01 (-0.05, 0.02) | -0.02 (-0.06, 0.01) | 0.01 (-0.04, 0.05) |
| Emotional Support | Cox | Mortality | 1.18 (0.93, 1.49) | **1.45 (1.05, 2.08)** | 1.03 (0.72, 1.49) |
|  |  |  |  |  |  |
| Emotional support * Time | Longitudinal | Verbal Episodic Memory | **-0.03 (-0.05, -0.01)** | -0.01 (-0.02, 0.00) | -0.04 (-0.09, 0.01) |
| Emotional Support | Cox | Mortality | 1.18 (0.93, 1.49) | **1.46 (1.03, 2.07)** | 0.99 (0.70, 1.42) |

*Note.* Linear mixed effect models were adjusted for age, interview mode, sex/gender, race and ethnicity, education levels, marital status, and depressive symptoms. The Executive function and verbal episodic memory scores were standardized to baseline mean and SD. Bolded estimates indicate *p* < 0.05.
